# **Robotic-assisted versus laparoscopic rectal surgery in obese and morbidly obese patients**: **ACS-NSQIP analysis**

**DOI:** 10.1007/s11701-022-01462-1

**Published:** 2022-10-21

**Authors:** Sinan Albayati, Kerry Hitos, Christophe R. Berney, Matthew J. Morgan, Nimalan Pathma-Nathan, Toufic El-Khoury, Arthur Richardson, Daniel I. Chu, Jamie Cannon, Greg Kennedy, James Wei Tatt Toh

**Affiliations:** 1 Department of Surgery, Nepean Hospital, Sydney, NSW Australia; 2 South Western Sydney Clinical School, University of New South Wales, Sydney, NSW Australia; 3 Discipline of Surgery, Sydney Medical School, University of Sydney, Sydney, NSW Australia; 4 Westmead Research Centre for Evaluation of Surgical Outcomes, Department of Surgery, Westmead Hospital, Westmead, NSW 2145 Australia; 5 Department of Surgery, Bankstown-Lidcombe Hospital, Sydney, NSW Australia; 6 Department of Surgery, Westmead Hospital, Sydney, NSW Australia; 7 Division of Gastrointestinal Surgery, University of Alabama, Birmingham, AL USA

**Keywords:** Robotic rectal surgery, Laparoscopic rectal surgery, Anterior resection, Cancer surgery, Obesity, Morbid obesity, Conversion to open

## Abstract

Laparoscopic rectal surgery within the confines of a narrow pelvis may be associated with a high rate of open conversion. In the obese and morbidly obese patient, the complexity of laparoscopic surgery increases substantially. Robotic technology is known to reduce the risk of conversion, but it is unclear if it can overcome the technical challenges associated with obesity. The ACS NSQIP database was used to identify obese patients who underwent elective laparoscopic or robotic-assisted rectal resection from 2015 to 2016. Obesity was defined as a body mass index (BMI) greater than or equal to 30 kg/m^2^. Morbid obesity was defined as a BMI greater than or equal to 35 kg/m^2^. The primary outcome was unplanned conversions to open. Other outcomes measures assessed included anastomotic leak, operative time, surgical site infections, length of hospital stay, readmissions and mortality. Statistical analyses were performed using SPSS 22.0 (IBM SPSS, USA). 1490 patients had robotic-assisted and 4967 patients had laparoscopic rectal resections between 2015 and 2016. Of those patients, 561 obese patients had robotic-assisted rectal resections and 1824 patients underwent laparoscopic rectal surgery. In the obese cohort, the rate of unplanned conversion to open in the robotic group was 14% compared to 24% in the laparoscopic group (*P* < 0.0001). Median operative time was significantly longer in the robotic group (248 min vs. 215 min, *P* < 0.0001). There was no difference in anastomotic leak or systemic sepsis between the laparoscopic and robotic rectal surgery groups. In morbidly obese patients (BMI ≥ 35 kg/m^2^), the rate of unplanned conversion to open in the robotic group was 19% compared to 26% in the laparoscopic group (*P* < 0.027). There was no difference in anastomotic leak, systemic sepsis or surgical site infection rates between robotic and laparoscopic rectal resection. Multivariate analysis showed that robotic-assisted surgery was associated with fewer unplanned conversions to open (OR 0.28, *P* < 0.0001). Robotic-assisted surgery is associated with a decreased risk of conversion to open in obese and morbidly obese patients when compared to conventional laparoscopic surgery. However, robotic surgery was associated with longer operative time and despite improvement in the rate of conversion to open, there was no difference in complications or length of stay. Our findings are limited by the retrospective non-randomised nature of the study, demographic differences between the two groups, and the likely difference in surgeon experience between the two groups. Large randomised controlled studies are needed to further explore the role of robotic rectal surgery in obese and morbidly obese patients.

## Introduction

The rate of obesity continues to increase and is a significant public health issue worldwide. Studies have observed an increase in morbidity, wound complications and longer hospital stay associated with obesity in patients undergoing laparoscopic and open colorectal resections [1–3].

Over the past 2 decades, minimally invasive surgery has become the standard of care in colorectal surgery, as it has been associated with reduced pain, length of stay and wound complications. However, laparoscopic colorectal surgery can be challenging in the obese patient due to limited space and access to deeper and narrow areas such as in the pelvis, and as a result of excess intra-peritoneal adiposity. Obesity has been shown to be associated with increased conversion to open rate, operative time, wound complications, and length of hospital stay [4–6]. This increased risk is worse with morbid obesity where the BMI is 35 or higher and ‘extreme’ or ‘severe’ obesity where the BMI is 40 or higher [4]. Operating in the confines of the narrow pelvis in rectal surgery adds further challenges to laparoscopic rectal surgery.

The technical advantages robotic surgery provides with superior views, improved instrument articulation and improved dexterity may offer the answers to the technical challenges of obesity in laparoscopic surgery. The ROLARR study is the largest RCT to date that compared robotic and laparoscopic rectal resections [7]. It showed no difference in conversion to open between robotic and laparoscopic surgery (8 vs 12%, *P* = 0.16) [7]. However, since then, two meta-analyses of eight RCTs (including the ROLARR study) found that robotic rectal resections were associated with fewer conversion to open compared to laparoscopic surgery [8, 9]. Other short-term surgical outcomes were similar with the two platforms.

The impact of BMI on robotic colorectal surgery was assessed in two observational studies [10, 11]. With the exception of longer operative time in obese patients, BMI did not seem to influence conversion to open, complication rates, length of hospital stay or readmissions associated with robotic colorectal surgery. Robotic colorectal surgery in obese patients (BMI > 30) was compared to conventional laparoscopy in two studies [12, 13]. Both studies showed robotic surgery was associated with longer time but shorter length of hospital stay compared to conventional laparoscopy. However, in these studies, there were no differences in conversion to open, complications rate or anastomotic leak. Those studies have been limited by their small sample size, which may explain the lack of statistical significance in many of the surgical outcomes measured.

As such, this study sought to interrogate the American College of Surgeons National Surgical Quality Improvement Project (ACS-NSQIP) database to evaluate if there is any advantage in using the robotic platform in obese patients for rectal resections, with the primary endpoint looking at unplanned conversion to open and secondary outcomes including complications, operative time, length of stay, readmission and mortality rates.

## Materials and methods

The American College of Surgeons National Surgical Quality Improvement Program (ASC NSQIP) participant use data files (PUF) for the years 2015 and 2016 were used to identify patients who underwent rectal resection procedures.

### Patient selection

All obese patients who underwent elective laparoscopic or robotic-assisted rectal resection were included in the study. Current Procedural Terminology (CPT) codes used were 44,145, 44,146, 44,207, 44,208. Obesity was defined as a BMI greater than or equal to 30 kg/m^2^ and morbid obesity was defined as a BMI greater than or equal to 35 kg/m^2^.

Patients were separated into laparoscopic rectal resection versus robotic-assisted rectal resection groups. The procedure was considered laparoscopic if it was coded laparoscopic or laparoscopic with unplanned conversion to open. The procedure was considered robotic-assisted if it was coded robotic or robotic with unplanned conversion to open.

### Outcomes measured

Primary outcome was unplanned conversions to open. Other outcomes measured included patient demographics, anastomotic leak, operative time, surgical site infections, length of hospital stay, readmission and mortality rates.

### Statistical analysis

Statistical analyses were performed using SPSS 22.0 (IBM SPSS, USA). Both univariable and multivariable (adjusted) logistic regression analyses were completed, including a subset analysis in patients who were morbidly obese (BMI ≥ 35 kg/m^2^).

## Results

A total of 1490 patients had robotic-assisted and 4967 patients underwent laparoscopic rectal resections between 2015 and 2016. Of those patients, 561 obese patients (37.65%) had robotic-assisted rectal resections and 1824 patients (36.7%) had laparoscopic rectal resections. Table 1 shows baseline characteristics of obese patients undergoing laparoscopic and robotic rectal resections. Age was similar between the two groups. More males compared to females had robotic rectal resection, but this did not reach statistical significance.

**Table 1:** Patient demographics and baseline characteristics of patients undergoing laparoscopic and robotic-assisted rectal resections

	LS *n* = 1824	RS *n* = 561	*P* value
Age (median (IQR))	58 (50–67)	57 (50–65)	0.079
Male (%)	913 (50%)	307 (55%)	0.05
Indications			
Acute diverticulitis	169	29	
IBD	16	2	
Cancer	814	326	
Other	825	204	
ASA			
1 or 2	407	107	0.1
3 or 4	479	148	0.1
Mechanical bowel preparation, *n* (%)	1173 (64%)	422 (75%)	0.001
Preoperative oral antibiotics, *n* (%)	752 (41%)	325 (60%)	0.001
Chemotherapy within 90 days, *n* (%)	122 (7%)	79 (14%)	0.0001
Stoma, *n* (%)	157 (9%)	141 (25%)	0.0001
Use of steroid/immunosuppressant	33 (1.8%)	4 (0.7%)	0.63
Diabetes	367 (20%)	109 (19.4%)	0.72
Current smoker	239 (13%)	80 (14%)	0.48
Dependent functional status	17 (0.9%)	6 (1%)	0.77
COPD	60 (3.3%)	15 (2.7%)	0.46
Congestive heart failure	10 (0.5%)	0	0.08
Hypertension	1000 (55%)	307 (55%)	0.97
Disseminated cancer	56 (3%)	29 (5%)	0.02
Steroid use for chronic condition	26 (3%)	8 (1.4%)	0.04
> 10% body weight loss in last 6 months	30 (1.6%)	6 (1%)	0.33

*LS* laparoscopic surgery, *RS* robotic-assisted surgery, *IQR* interquartile range, *IBD* inflammatory bowel disease.

Patients in the robotic group were more likely to have had mechanical bowel preparation and preoperative oral antibiotics before surgery and chemotherapy within 90 of surgery. There were also more likely to have disseminated cancer at the time of surgery and more likely to have a stoma.

Table 2 shows operative outcomes of obese patients (BMI ≥ 30) undergoing laparoscopic and robotic-assisted rectal resections. The rate of unplanned conversion to open in the robotic group was 14% compared to 24% in the laparoscopic group (*P* < 0.0001). Median operative time was longer in the robotic group (248 min vs. 215 min, *P* < 0.0001). There was no difference in anastomotic leak rate or systemic sepsis between laparoscopic and robotic rectal resections. The overall rate of surgical site infection was similar in both groups. However, organ space surgical site infection was more common following robotic rectal resection (5.3 vs. 3.5%, *P* = 0.04).

**Table 2:** Operative outcomes of obese patients (BMI ≥ 30) undergoing laparoscopic and robotic-assisted rectal resections

	LS *n* = 1824	RS *n* = 561	*P* value
Unplanned conversion	435 (24%)	79 (14%)	< 0.0001
Anastomotic leak	56 (3%)	24 (4%)	0.2
Operative time, median (IQR)	215 (160–284)	248 (196–323)	< 0.0001
Transfusion ≥ 1unit	4 (0.2%)	1 (0.18%)	0.085
Systemic sepsis	4 (0.22%)	0	0.27
SSI	171 (9%)	60 (10%)	0.45
Superficial SSI	103 (5.6%)	31 (5.5%)	0.9
Deep SSI	15 (0.8%)	3 (0.5%)	0.5
Organ space SSI	63 (3.5%)	30 (5.3%)	0.04
Prolonged NGT use	175 (9.6%)	60 (10.7%)	0.45
Pneumonia	10 (0.55%)	4 (0.7%)	0.65
Acute myocardial infarction	10 (0.55%)	3 (0.5%)	0.1
Unplanned reoperation	70 (4%)	27 (5%)	0.33
Unplanned readmission	161 (9%)	60 (11%)	0.18
LOHS, median (IQR)	4 (3–6)	4 (3–5)	0.027

*LS* laparoscopic surgery, *RS* robotic-assisted surgery, *IQR* interquartile range, *SSI* surgical site infection, *NGT* nasogastric tube, *LOHS* length of hospital stay.

In morbidly obese patients (BMI ≥ 35 kg/m^2^), the rate of unplanned conversion to open in the robotic group was 19% compared to 26% in the laparoscopic group (*P* < 0.027). Similarly, there was no difference in anastomotic leak, systemic sepsis or surgical site infection rates between robotic and laparoscopic rectal resection (Table 3).

**Table 3:** Operative outcomes of morbidly obese patients (BMI ≥ 35) undergoing laparoscopic and robotic-assisted rectal resections

	LS *n* = 728	RS *n* = 241	*P* value
Unplanned conversion	187 (26%)	45 (19%)	0.027
Anastomotic leak	26 (4%)	13 (5%)	0.22
Systemic sepsis	2 (0.3%)	1 (0.4%)	0.7
Superficial SSI	51 (7%)	11 (4.6%)	0.18
Deep SSI	6 (0.8%)	1 (0.4)	0.5
Organ space SSI	30 (4%)	13 (5%)	0.4
Pneumonia	5 (0.7%)	1 (0.4%)	0.64
Myocardial infarction	6 (0.8%)	2 (0.8%)	0.22
Return to theatre	36 (4.9%)	14 (5.8%)	0.6
Readmission	77 (10.6%)	26 (10.7%)	0.9

*LS* laparoscopic surgery, *RS* robotic-assisted surgery, *SSI* surgical site infection.

Table 4 shows univariate and multivariate logistic regression analysis of predictors of unplanned conversion to open following laparoscopic and robotic-assisted rectal resections. Multivariate analysis showed that robotic-assisted surgery was associated with fewer unplanned conversions to open (OR 0.28, *P* < 0.0001). Other predictors of increased rate of unplanned conversion to open include male gender, ASA 3 or more, preoperative weight loss, extended operative time, and hypertension.

**Table 4:** Univariate and adjusted multivariate analysis of demographics, medical comorbidities, surgical approach and peri-operative variables predicting unplanned conversion to open in obese patients undergoing rectal resection

Variable	Adjusted OR (95% CI)	*P* value	Univariate OR (95% CI)	*P* value
Age ≥ 80 years	1.43 (1.11–1.85)	0.006	1.19 (0.69–2.05)	0.529
Male	1.07 (0.94–1.22)	0.280	1.29 (1.00–1.67)	0.049
Indication				
Acute diverticulitis	(Ref.)			
Crohn’s/ulcerative colitis	1.97 (1.11–3.49)	0.020	2.13 (0.60–7.47)	0.240
Colorectal cancer	0.87 (0.68–1.12)	0.272	0.84 (0.51–1.38)	0.480
Other	1.16 (0.90–1.48)	0.259	1.27 (0.78–2.07)	0.333
Stoma	1.63 (1.32–2.01)	<0.0001	2.31 (1.50–3.57)	<0.0001
Chemotherapy	0.91 (0.75–1.10)	0.317	0.87 (0.59-1.30)	0.499
Steroid/immunosuppressant	1.49 (0.99–2.25)	0.062	0.96 (0.32–2.90)	0.940
Diabetes	1.13 (0.94–1.36)	0.189	0.88 (0.61-1.26)	0.472
Current smoker	1.14 (0.96–1.35)	0.140	0.91 (0.65–1.30)	0.613
Dyspnoea	1.66 (1.24–2.23)	0.001	1.43 (0.77–2.65)	0.258
Functional status				
Independent	(Ref.)			
Dependent	1.14 (0.62–2.11)	0.666	0.18 (0.04–0.86)	0.860
Unknown	0.41 (0.20–0.85)	0.017	0.42 (0.09–1.86)	0.255
History of COPD	1.25 (0.89–1.75)	0.187	0.79 (0.39–1.63)	0.532
Ascites	4.62 (0.65–32.84)	0.126	1.88 (0.23–15.29)	0.999
Congestive heart failure	0.87 (0.25–2.97)	0.818	–	–
Hypertension	1.30 (1.15–1.48)	<0.0001	1.41 (1.08–1.84)	0.011
Disseminated Cancer	1.21 (0.90–1.62)	0.201	1.34 (0.80-2.24)	0.272
Wound infection	1.65 (0.80–3.41)	0.174	–	–
Steroid use	1.88 (1.37–2.57)	<0.0001	0.85 (0.40–1.82)	0.681
Weight loss	2.21 (1.59–3.10)	<0.0001	2.82 (1.46-5.45)	0.002
Bleeding disorders	1.93 (1.27–2.90)	0.002	1.51 (0.61–3.78)	0.375
Transfusion ≥1U PRBC (72hrs)	4.63 (1.34–16.02)	0.016	4.26 (0.55–32.76)	0.164
Systemic sepsis (before surgery)	1.04 (0.46–2.37)	0.921	–	–
Albumin (≥3.50)	0.414 (0.30–0.57)	<0.0001	0.43 (0.30–0.61)	<0.0001
Haematocrit (≥30%)	0.66 (0.41–1.01)	0.106	1.03 (0.53–2.0)	0.928
ASA ≥3	1.39 (1.16–1.67)	<0.0001	1.31 (1.00–1.72)	<0.0001
Operative time (≥180 min)	2.23 (1.92–2.59)	<0.0001	2.07 (1.55–2.75)	<0.0001
Approach (robotic)	0.43 (0.36–0.52)	<0.0001	0.28 (0.19–0.41)	<0.0001

## Discussion

Laparoscopic surgery has become the standard of care in colorectal surgery. However, laparoscopic surgery can be challenging when operating with straight and rigid instruments in the confines of the narrow pelvis. Furthermore, there is still concern about the safety of laparoscopic rectal surgery compared with open surgery. These concerns increased following the AlaCart and the ACOSOG Z6051 trials both of which failed to show non-inferiority of laparoscopic surgery to open surgery for pathological outcomes [14, 15].

The development of robotic assisted laparoscopic surgery with superior views, improved instrument articulation and enhanced dexterity has emerged as a potential solution to the limitations of conventional laparoscopic surgery. Despite this, the role of the robotic system in colorectal surgery is still debated, and its utility in obese and morbidly obese patients has been less than clear. To date, there has been sparse literature on whether the technical challenges associated with obesity may be improved by the technical advantages offered by robotic surgery, or whether obesity may hinder the robotic platform due to the difficulties of port placement, docking and arm collisions.

In this study, we compared 561 patients who had robotic assisted rectal resection with 1824 patients who had conventional laparoscopy using data from the ACS-NSQIP database. The unplanned conversion to open following laparoscopic surgery for obese patients was very high (24%). In comparison, the rate of conversion to open in previous studies that included experienced laparoscopic surgeons was much lower (9–12%) [7, 14, 15]. One explanation to this is that obesity represents a significant challenge to laparoscopic rectal surgery, especially within the confines of a narrow pelvis in a morbidly or ‘extremely’ obese patient. Another explanation is that the results of this data come from surgeons with varying experience in laparoscopic surgery.

Overall, robotic surgery significantly reduced the rate of unplanned conversion to open compared to conventional laparoscopy (14 vs 24%, *P* < 0.0001) but was associated with longer operative time (248 min vs 215 min, *P* < 0.0001). There was no significant difference in systemic sepsis, surgical site infection or length of hospital stay. When looking at the subset of morbidly obese patients (BMI 35 or greater), the reduction in the rate of conversion to open was also significantly lower in the robotic group. This demonstrates that the benefits of robotic technology with its articulating wrists, 3D vision and surgeon-controlled robotic arms can still counter the challenges associated with not only obese but also morbidly obese patients. A sub-analysis for ‘extreme’ obesity was not performed due to small numbers in this group.

Multivariate logistic regression analysis of variables predicting unplanned conversion to open showed that robotic-assisted surgery was associated with fewer unplanned conversions to open with adjusted odds ratio 0.28. Other predictors of increased rate of unplanned conversion to open included male gender, ASA 3 or more, preoperative weight loss, prolonged operative time, stoma formation and hypertension. Previous studies have identified male sex, advanced tumour stage, and hypertension as additional risk factors for unplanned conversion to open in both laparoscopic and robotic colorectal surgery [16–18]. Preoperative weight loss and the formation of stoma could be indicators for more advanced disease, and this could explain their association with increased risk of conversion to open. The evidence to support ASA as a predictor of conversion to open is conflicting. While some studies identified ASA of 3 or more as a predictor of unplanned conversion to open, other studies failed to confirm this [16, 19].

Robotic assisted rectal resection has been compared to laparoscopy in four retrospective studies using data from the ASC-NSQIP [20–23]. All studies showed reduction in the rate of unplanned conversion to open associated with robotic surgery and three of them reported reduced length of hospital stay with robotic surgery [20–22]. The rate of post-operative complications including anastomotic leak and surgical site infections was the same following both laparoscopic and robotic rectal resections. Ahmed et al. [24] prospectively compared laparoscopic and robotic rectal resection in high-risk patients. High risk factors included male gender, obesity (BMI > 30), preoperative chemoradiation, tumour lower than 8 cm from the anal verge and previous abdominal surgery. In 184 high-risk patients (99 robotic surgery and 85 laparoscopic surgery), robotic surgery was associated with fewer conversions to open (0 vs 5%, *P* = 0.043), shorter hospital stay (7 vs 9 days, *P* = 0.001) and higher sphincter preservation rate (86 vs 74%, *P* = 0.045).

Two studies compared robotic and laparoscopic rectal resections in obese patients (BMI > 30) [12, 13]. Panteleimonitis et al. [12] retrospectively matched 63 patients with robotic rectal resection with 61 patients who had laparoscopic surgery. As expected, operative time was longer in the robotic group compared to conventional laparoscopy (260 min vs 215 min, *P* = 0.0001). However, length of hospital stay was shorter in the robotic group (6 vs 8 days, *P* = 0.014) and 30-day readmission rate was lower (6.3 vs 19.7%, *P* = 0.033). On the other hand, there was no difference in the rate of post-operative complications, anastomotic leak, lymph node yield or Ro clearance between the two groups. Although conversion to open surgery was fewer with robotic surgery, this did not reach statistical significance (0 vs 3.3%, *P* = 0.24). Gorgun et al. [13] retrospectively compared 29 patients who had robotic rectal surgery with 27 patients who had laparoscopic surgery. Like the previous study, there were fewer conversions to open with robotic surgery, but this did not reach statistical significance (3.4 vs 18.5%, *P* = 0.09). We may assume that this result was underpowered due to small sample size. Return of bowel function was faster following robotic surgery (3 days vs 4 days, *P* = 0.01), and hospital stay was shorter (6 days vs 7 days, *P* = 0.02). There was no difference in the rate of postoperative complications including anastomotic leak and surgical site infection between robotic and laparoscopic surgery. There was also no difference between completeness of mesorectal fascia excision or lymph node yield between the two groups.

Shiomi et al. compared short-term outcomes of robotic versus laparoscopic rectal cancer resection in obese patients defined by visceral fat area of ≥ 130 cm^2^, assessed by computer tomography (CT) [25]. They compared 52 patients who had robotic rectal surgery with 30 patients who underwent conventional laparoscopy. There were no conversions to open in both groups. Overall complication rate was significantly lower in the robotic group (9.6 vs. 30%, *P* = 0.04) and there was shorter hospital stay following robotic surgery compared to laparoscopy (7 days vs 9 days, *P* = 0.001).

Our study has several limitations. The study is a retrospective analysis of a prospectively maintained database which makes it prone to inclusion bias. In this study, more patients in the robotic group had mechanical bowel preparation, received preoperative oral antibiotics and had a formation of stoma. Although these do not directly impact on the rate of conversion to open, these peri-operative interventions may influence many of the post-operative outcomes including anastomotic leak, systemic sepsis and surgical site infection. There were also differences in patient demographics, lack of standardization of surgical technique and definition of conversion to open, inclusion of outcomes from low volume centers as well as surgeons with different levels of expertise in both laparoscopic and robotic colorectal surgery.

## Conclusions

Robotic-assisted rectal surgery is associated with a decreased risk of conversion to open overall, as well as in obese and morbidly obese patients when compared to conventional laparoscopic rectal surgery. However, robotic surgery is associated with longer operative time and despite improvement in the rate of conversion to open, there was no difference in the rate of anastomotic leak, systemic sepsis, overall surgical site infection or length of hospital stay. Large randomised controlled studies are needed to further explore the role of robotic rectal surgery in obese, morbidly obese and extremely obese patients.
